# Glial Triad in Diabetic Neuropathy: Central Players in Neuropathic Pain Pathogenesis and Disease-Modifying Therapeutic Avenues

**DOI:** 10.3390/biomedicines14020435

**Published:** 2026-02-14

**Authors:** Siyu Fu, Yaoyao Guo, Mengke Cheng, Huiyan Duan, Qiongyao He, Huihui Ren, Gang Yuan

**Affiliations:** Department of Endocrinology, Tongji Hospital, Tongji Medical College, Huazhong University of Science & Technology, No. 1095 Jiefang Avenue, Wuhan 430030, China; fusiyu52@outlook.com (S.F.); guoyaoyao97@163.com (Y.G.); d202582666@hust.edu.cn (M.C.); duan54290@163.com (H.D.); heqiongyao1126@163.com (Q.H.)

**Keywords:** diabetic neuropathy, neuropathic pain, glial cells, microglia, astrocytes, neuroinflammation, central sensitization, biomarkers

## Abstract

Painful diabetic neuropathy (PDN) is a prevalent and debilitating complication of diabetes, characterized by persistent neuropathic pain that severely impairs quality of life. Current management strategies predominantly target peripheral nerve dysfunction and offer only symptomatic relief, with no disease-modifying therapies available. Emerging evidence now underscores the critical role of central nervous system (CNS) glial cells—microglia, astrocytes, and oligodendrocytes, collectively termed the “glial triad”—in driving PDN pathogenesis. This review synthesizes recent advances elucidating how these glial cells contribute to neuroinflammation, metabolic dysregulation, and central sensitization. We detail specific mechanisms including microglial *NLR Family Pyrin Domain Containing 3* (*NLRP3*) inflammasome activation and metabolic reprogramming, astrocytic aquaporin-4 (*AQP4*) polarity disruption impairing glymphatic function, and oligodendrocyte myelination deficits via Mammalian Target of Rapamycin (*mTOR*) signaling. Furthermore, we discuss the translational potential of glia-derived biomarkers (e.g., Translocator Protein (*TSPO*), Glial Fibrillary Acidic Protein (*GFAP*), myelin basic protein (*MBP*)) for early diagnosis and patient stratification. Finally, we highlight promising therapeutic avenues that target glial pathways, such as interleukin-35 (IL-35), β-hydroxybutyrate, and metformin, which aim to shift the treatment paradigm from symptomatic control to disease modification. By integrating preclinical and clinical insights, this review proposes the glial triad as a central player in PDN and suggests that targeted glial interventions may represent a promising frontier for future disease-modifying strategies.

## 1. Introduction

Diabetes mellitus (DM) is a metabolic disorder characterized by chronic hyperglycemia, classified into type 1 diabetes (T1DM) and type 2 diabetes (T2DM) [1]. Prolonged hyperglycemia in diabetes leads to multi-organ damage, with the nervous system being particularly vulnerable [2]. Diabetic neuropathy (DN), one of the most prevalent complications of diabetes, affects approximately 50% of individuals with diabetes [3], manifesting in heterogeneous clinical subtypes. These include peripheral neuropathy, autonomic neuropathy, and proximal neuropathy [4], among others. Painful diabetic neuropathy (PDN) represents a predominant clinical phenotype, distinguished by debilitating neuropathic pain symptoms such as spontaneous burning sensations, lancinating pain, and mechanical allodynia, which severely impair quality of life [5].

PDN is a chronic neurological disorder predominantly driven by peripheral nerve injury, clinically characterized by burning dysesthesia, lancinating pain, and mechanical allodynia [6]. In severe cases, PDN progresses to functional disability, profoundly impairing patients’ quality of life [7]. Typically emerging in the advanced stages of diabetes, PDN is strongly associated with prolonged hyperglycemic exposure and suboptimal glycemic control [8]. Affected individuals frequently exhibit sensory abnormalities (e.g., hypoesthesia or paresthesia) and motor deficits (e.g., muscle weakness), with symptom severity escalating in parallel with disease chronicity [9].

The hallmark manifestations of PDN encompass bilateral distal limb involvement, featuring nocturnal exacerbation of burning sensations, numbness, and paroxysmal stabbing pain [10]. Epidemiological studies indicate that approximately 26% of diabetic patients develop PDN, with a disproportionate prevalence among those with T2DM [11]. Against the backdrop of the global diabetes pandemic, PDN has emerged as a critical public health challenge, imposing substantial socioeconomic burdens [12]. Beyond its physical morbidity, PDN is intricately linked to neuropsychiatric comorbidities, including sleep disturbances, anxiety, and depression, which collectively compromise psychosocial functioning and societal participation [13].

Emerging epidemiological data underscore a positive correlation between the duration of diabetes and the incidence of diabetic neuropathy [14]. In T2DM, the prevalence of PDN escalates markedly after 10–15 years of disease progression [15]. The pathogenesis of PDN is multifactorial, driven by chronic hyperglycemia-induced neuroaxonal degeneration, oxidative stress, and neuroimmune dysregulation [16]. These mechanisms synergistically perpetuate peripheral and central sensitization, fostering a self-reinforcing cycle of neuroinflammation and nociceptive signaling [17].

While significant advances have been made in elucidating peripheral mechanisms of diabetic neuropathy—including axonal injury, inflammatory cascades, and neurotrophic factor depletion [18]—the role of central nervous system (CNS) glial cells in PDN remains underexplored [19]. Glial populations (microglia, astrocytes, and oligodendrocytes) constitute dynamic regulators of CNS homeostasis, yet their reactive phenotypes and functional perturbations in PDN are poorly characterized [20].

Microglia, the resident immune sentinels of the CNS, exhibit pathological hyperactivation under diabetic conditions, potentially amplifying pain processing via maladaptive neuroimmune crosstalk [21]. Astrocytes, critical for synaptic glutamate clearance and metabolic support, undergo reactive gliosis in diabetes, which may exacerbate neuronal excitability and sustain central sensitization [22]. Oligodendrocyte dysfunction, implicated in myelination deficits, could further disrupt pain-modulatory pathways [23]. Despite preclinical evidence linking glial activation to neuropathic pain states, mechanistic insights into their spatiotemporal contributions to PDN are fragmentary [24].

The paucity of CNS-focused PDN research represents a critical knowledge gap [25]. Current biomarker discovery efforts predominantly target peripheral nerve pathology, neglecting glia-derived mediators such as CSF-soluble *TREM2* (microglial activity) [26], *AQP4* polarity indices (astrocyte metabolic function) [27], or myelin integrity markers (oligodendrocyte health) [28]. Unlocking the therapeutic potential of glial modulation may offer a promising new avenue to advance PDN management, with the potential to shift the treatment paradigm from symptomatic relief toward disease modification [29].

**Figure 1:**
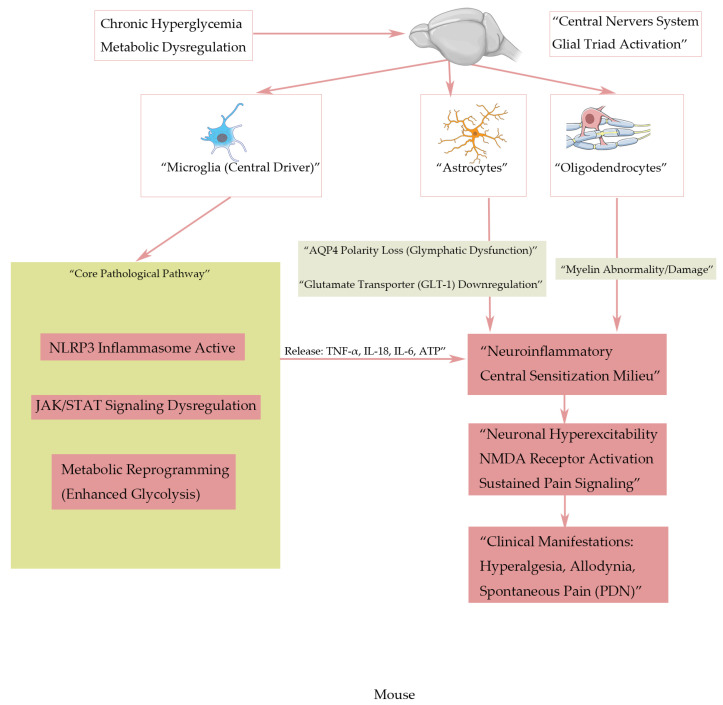
Integrated pathogenic mechanism of the glial triad in PDN. This schematic illustrates how chronic hyperglycemia and metabolic dysregulation drive the maladaptive activation and crosstalk of the central glial triad (microglia, astrocytes, and oligodendrocytes), culminating in central sensitization and neuropathic pain. Hyperglycemia initiates widespread glial activation in the spinal cord. Microglia act as the central inflammatory hub, undergoing activation through parallel pathways including *NLRP3* inflammasome formation, dysregulated *JAK/STAT* signaling, and metabolic reprogramming (e.g., enhanced aerobic glycolysis). This activated state leads to sustained release of pro-inflammatory mediators (Tumor Necrosis Factor-α (*TNF-α*), Interleukin-1β (*IL-1β*), Interleukin-6 (*IL-6*)) and danger signals (e.g., adenosine triphosphate (ATP)). These microglial outputs directly disrupt the function of neighboring astrocytes and oligodendrocytes. Astrocytes develop loss of *AQP4* polarity (impairing glymphatic clearance), dysfunctional connexin 43 (*Cx43*) hemichannels, and downregulation of the glutamate transporter glutamate transporter 1 (*GLT-1*). Oligodendrocytes exhibit myelin dysfunction and impaired integrity. Collectively, these glial disturbances create a self-reinforcing microenvironment characterized by neuroinflammation, excitotoxicity (due to glutamate accumulation), and disrupted metabolic support. This pathological milieu converges on spinal cord neurons, promoting excessive N-Methyl-D-Aspartate (*NMDA*) receptor activation, increased intracellular Ca^2+^, and transcriptional changes that underlie central sensitization—the key neural mechanism responsible for the clinical symptoms of PDN, including hyperalgesia, allodynia, and spontaneous pain. The model underscores that PDN is perpetuated not by isolated glial dysfunction, but by the maladaptive interactions within the glial triad.

This review aims to elucidate the multifaceted roles of glial cells within the central nervous system (CNS) in the pathogenesis of painful diabetic neuropathy (PDN), with a focused analysis on microglia, astrocytes, and oligodendrocytes as key modulators of nociceptive processing, neuroinflammatory cascades, and neurorestorative processes [30]. By systematically reviewing preclinical and clinical evidence, we critically evaluate the mechanistic contributions of these glial subsets to PDN progression, synthesizing their molecular signatures (e.g., *TSPO* for microglial activation, *AQP4* polarity for astrocytic dysfunction, *MBP* for oligodendrocyte integrity) and translational relevance [31]. Special emphasis is placed on identifying glia-derived biomarkers with potential utility in early PDN diagnosis, disease stratification, and therapeutic targeting [32] (Figure 1).

## 2. Methods

This article is a narrative review that systematically integrates and critically evaluates the existing evidence on the role of the glial triad in the pathogenesis and treatment of PDN. To ensure a comprehensive and representative synthesis, a structured approach to literature identification and selection was employed.

A systematic literature search was conducted across PubMed, Web of Science, and Google Scholar databases for publications up to December 2024. The search strategy utilized combinations of keywords and MeSH terms, including “painful diabetic neuropathy,” “diabetic neuropathic pain,” “glial cells,” “microglia,” “astrocyte,” “oligodendrocyte,” “neuroinflammation,” and “central sensitization.” Original research articles, reviews, and meta-analyses published in English from 2018 onward were included, with a focus on studies elucidating the mechanistic roles of central glial cells in established PDN preclinical models (e.g., STZ-induced, db/db) or providing relevant clinical insights. Studies focusing solely on peripheral neuropathy mechanisms, conference abstracts, and non-English publications were excluded.

It is important to clarify the methodological scope and inherent limitations of this review. As a narrative synthesis, its primary aim is conceptual integration and mechanistic hypothesis-building rather than quantitative data pooling. Therefore, while the search and selection process followed a systematic approach to ensure breadth, no formal quality assessment of individual studies (e.g., using specific risk-of-bias tools) or meta-analytic statistical pooling was performed. This approach prioritizes the construction of a coherent “glial triad” framework to explore interactions and pathways but acknowledges the potential for selection bias and does not provide a graded, quantitative summary of the evidence strength. Specific statistical results cited in the text are presented as reported in the original studies to illustrate findings within their specific experimental contexts.

## 3. Glial Cells: The Hidden Orchestrators of PDN Pathogenesis

As the primary immune surveillants of the CNS, microglia propel PDN progression through phase-specific activation patterns. Teneligliptin and Semaglutide alleviate diabetic neuropathic pain by suppressing spinal astrocyte and microglia activation, demonstrating the neuro-anti-inflammatory potential of GLP-1R signaling [33]. During the early phase (0–2 weeks), Toll-like receptor 4/myeloid differentiation primary response 88 (*TLR4/MyD88*)-dependent signaling triggers TNF-α/IL-1β release and hyperactivation of Mitogen-Activated Protein Kinase/Nuclear Factor Kappa-Light-Chain-Enhancer of Activated B Cells (*MAPK/NF-κB*) pathways, directly amplifying nociceptive transmission [34]. In the intermediate-to-late phases, microglial perpetuation of chronic inflammation is mediated by CX3C chemokine receptor 1 (*CX3CR1*)/*NLRP3* inflammasome axis activation [35] and metabolic reprogramming characterized by sirtuin 3 (Sirt3) downregulation and aerobic glycolysis (Warburg effect) [36], culminating in pyroptotic cell death (Gasdermin D (*GSDMD*)-mediated [37]). Therapeutic strategies to rebalance microglial states include phenotypic polarization (e.g., IL-35-induced M2 shift via Janus kinase 2/signal transducer and activator of transcription 6 (*JAK2/STAT6*) [38]) and metabolic modulation (e.g., metformin-mediated *Sirt3* restoration [36]), both demonstrating efficacy in preclinical neuroinflammation models.

Astrocytic dysfunction lies at the epicenter of PDN’s self-reinforcing “metabolo-inflammatory” cascade. Hyperglycemia-induced *AQP4* polarity disruption (via matrix metalloproteinase-9 (*MMP-9*)-mediated β-dystroglycan cleavage [39]) impairs glymphatic waste clearance, leading to glutamate excitotoxicity and *NMDA* receptor hyperactivation [40]. Concurrently, histone deacetylase 5/signal transducer and activator of transcription 3 (*HDAC5-STAT3*) axis dysregulation (impaired acetylation homeostasis [41]) and C-X-C motif chemokine ligand 13/C-X-C motif chemokine receptor 5 (*CXCL13/CXCR5*)-mediated chemotactic signaling [42] exacerbate neuroinflammatory cascades. Breaking this cycle requires interventions like *AQP4* repolarization (β-hydroxybutyrate-induced syntrophin alpha 1 (*SNTA1*)upregulation [43]) and gap junction restoration (lycopene-mediated Cx43 stabilization [44]), which collectively ameliorate astrocytic metabolic-immune cross-talk.

Emerging evidence implicates oligodendrocyte dysfunction in PDN through dual mechanisms: ① demyelination-induced Aδ fiber conduction abnormalities, exacerbated by adaptor protein, phosphotyrosine interacting with PH domain and leucine zipper 1 (APPL1) deficiency-driven mTOR/Ras-related protein Rab5 (Rab5) signaling hyperactivation [45]; ② oligodendrocyte precursor cell (OPC)-derived C-C Motif Chemokine Ligand 2/C-C Motif Chemokine Receptor 2 (*CCL2/CCR2*)axis activation in spinal dorsal horn, potentiating nociceptive neurotransmission [46]. While therapeutic exploration remains nascent, strategies targeting myelin regeneration (metformin-mediated mTOR modulation) or *APPL1/mTOR* pathway rebalancing show promise in preclinical models for mitigating central sensitization.

Beyond the cytokine network, glial cells and neurons also share key molecular sensors. For example, the Transient Receptor Potential Ankyrin 1 (*TRPA1*) channel is expressed not only in nociceptive neurons but also in astrocytes and oligodendrocytes. As a common downstream target for multiple algogenic substances, *TRPA1* may serve as a critical hub mediating glia-neuron crosstalk and amplifying central sensitization signals [47].

## 4. Microglia: Initiators of Inflammatory Storms in PDN

### 4.1. Inflammatory Pathways

#### 4.1.1. *NF-κB* Signaling Pathway

Spinal microglial activation is significantly augmented in PDN rat models. Immunofluorescence analysis revealed elevated protein levels of the microglial marker Ionized Calcium-Binding Adapter Molecule 1 (IBA-1) in diabetic spinal cords compared to controls (*p* < 0.01), correlating with increased pro-inflammatory cytokine release. Diabetic rats exhibited heightened TNF-α and IL-1β levels accompanied by phosphorylation of p38 mitogen-activated protein kinase (*p38*), Extracellular Signal-Regulated Kinase (*ERK*), and c-Jun N-terminal kinase (*JNK*) kinases-molecular mediators of hyperalgesia. Pharmacological inhibition of *p-p38* and *p-JNK* pathways by Ammoxetine attenuated microglial activation and cytokine accumulation, demonstrating their critical role in nociceptive regulation. Notably, reduced inhibitor of NF-kappaB alpha (IκBα) protein levels in diabetic spinal cords suggested *NF-κB/p65* pathway activation, establishing its pivotal involvement in peripheral inflammatory hypersensitivity [48].

*Sirtuin 3* (*Sirt3*), a mitochondrial deacetylase governing cellular metabolism and oxidative stress, showed hyperglycemia-induced downregulation during DNP progression. *Sirt3* deficiency exacerbated spinal dorsal horn (SDH) microglial activation (Iba-1+ cells) with concomitant upregulation of IL-6, IL-1β, and TNF-α mRNA (*p* < 0.05 vs. wild-type). Genetic ablation of *Sirt3* promoted *NF-κB* and *MAPK* pathway activation, whereas *Sirt3* overexpression in BV-2 microglia attenuated high glucose-induced Iba-1 expression and suppressed IL-1β/TNF-α production through *NF-κB/MAPK* inhibition [36].

Intrathecal delivery of transcription factors achaete-scute family bHLH transcription factor 1/LIM homeobox 6 (*Ascl1/Lhx6*) rebalanced cytokine profiles, reducing pro-inflammatory mediators (TNF-α, IL-1β) while elevating anti-inflammatory cytokines (IL-4, IL-10, IL-13). This intervention concurrently suppressed spinal *p38/JNK/NF-κB* activation, achieving 62% reduction in neuropathic pain behaviors [49].

#### 4.1.2. NLRP3 Inflammasome Axis

Intracerebroventricular administration of GLP-1 receptor agonists (*GLP-1RAs*) alleviated thermal hyperalgesia and mechanical allodynia in PDN rats through microglial *NLRP3* modulation. RNA sequencing demonstrated 3.8-fold *NLRP3* downregulation post-GLP-1RA treatment, with immunofluorescence co-localization showing 72% reduction in *NLRP3*/Iba-1 interactions. This mechanism effectively inhibited lipopolysaccharide (LPS)-induced inflammasome activation in microglia (*p* < 0.01 vs. vehicle) [50].

*TANK-binding kinase 1* (*TBK1*) emerged as a critical mediator of *NLRP3* inflammasome activation via non-canonical *NF-κB* signaling. Immunofluorescence localized *TBK1* predominantly to Iba1+ microglia versus GFAP+ astrocytes and NeuN+ neurons. *TBK1* silencing reduced microglial aggregation by 45% and pyroptosis markers (*GSDMD*) in PDN models. The *TBK1* inhibitor AMX attenuated neuroinflammation through dual inhibition of *NF-κB* activation and *NLRP3* inflammasome assembly [37].

#### 4.1.3. *TLR9/p38 MAPK* Signaling

In streptozotocin (STZ)-induced PDN models, Toll-like receptor 9 (*TLR9*) upregulation drove microglial activation via *p38 MAPK/ERK1/2* phosphorylation. *TLR9* knockdown using short hairpin RNA (shRNA) lentivirus attenuated inflammatory cytokine secretion (TNF-α↓, IL-1β↓, IL-6↓) and reversed microglial chemotaxis (IBA1+ cells↓). Intrathecal SB203580 (20 μM p38 inhibitor) produced comparable anti-nociceptive effects, validating *TLR9*’s pathogenic role through *p38*-dependent mechanisms [51].

#### 4.1.4. *JAK-STAT* Signaling Pathway

Interleukin-35 (IL-35) exhibits therapeutic potential in diabetic neuropathic pain (DNP) by modulating microglial polarization. In DNP rat models, IL-35 administration significantly elevated mechanical withdrawal thresholds (MWT) and thermal withdrawal latencies (TWL), concurrent with activation of the anti-inflammatory *JAK2/STAT6* pathway. In LPS-stimulated microglia, IL-35 suppressed *JNK* phosphorylation while enhancing *JAK2/STAT6* signaling, leading to upregulated expression of Arginase-1 (Arg-1) and Interleukin-10 (IL-10). These findings establish IL-35 as a potent activator of the *JAK2/STAT6* cascade in pain regulation [38].

Jinmaitong (JMT) alleviates neuroinflammation through *JAK2/STAT3* inhibition. The diabetes model was constructed using 11 to 12-week-old male Zucker diabetic fatty (ZDF) rat (fa/fa). DNP rats exhibited elevated phosphorylation levels of *JAK2* and *STAT3* in spinal microglia, which were normalized to 89% and 93% of control values respectively following JMT treatment (*p* < 0.05). This pharmacological intervention correlated with reduced microglial activation and attenuated neuropathic pain behaviors [52].

Immunofluorescence co-localization revealed predominant p-STAT3 expression in activated microglia within the dorsal horn. Intrathecal administration of the *JAK2* inhibitor AG490 from day 3 post-modeling reduced p-JAK2 and p-STAT3 expression over a 14-day period (*p* < 0.01). In vitro studies demonstrated that AG490 suppressed high glucose-induced p-STAT3 activation in microglia, subsequently decreasing neuronal p-caveolin-1 (p-CAV-1) and p-NMDA receptor subunit 2B (p-NR2B) in co-culture systems. These data implicate the microglial *JAK2/STAT3-CAV1-NR2B* axis as a critical mediator of diabetic neuropathic pain pathogenesis [53].

#### 4.1.5. P2 Receptor Pathways

P2Y purinoceptor 14 (*P2Y14*) receptor overexpression exacerbates microglial activation and neuroinflammation in PDN models. *P2Y14* shRNA intervention achieved 68% receptor knockdown (*p* < 0.001), accompanied by reduced microglial activation, suppressed TNF-α and IL-1β levels, and inhibited *p38 MAPK* phosphorylation. Mechanistically, long non-coding RNA (LncRNA)-UC.25 + shRNA enhances signal transducer and activator of transcription 1 (*STAT1*) binding to *P2Y14* promoter regions, thereby downregulating *P2Y14* expression and mitigating neuropathic pain [54].

In STZ-induced diabetic models, spinal *P2X7 receptor* (*P2X7R*) expression increased, predominantly localized to Iba1+ microglia in the dorsal horn. Pharmacological antagonism or genetic ablation of *P2X7R* attenuated mechanical allodynia progression, with no detectable co-localization observed with GFAP+ astrocytes or NeuN+ neurons. This microglia-specific *P2X7R* signaling represents a novel therapeutic target for diabetic neuropathic pain management [55].

#### 4.1.6. Microglial Phenotypic Switching

Microglial polarization plays a dual role in painful diabetic neuropathy (PDN), transitioning between pro-inflammatory M1 (inducible nitric oxide synthase (iNOS+), TNF-α+) and anti-inflammatory M2 (cluster of differentiation 206 (CD206+), Arg-1+) phenotypes. Diabetic mice exhibited 62% reduction in spinal Insulin-Like Growth Factor 1 (IGF-1) levels (*p* < 0.001), concomitant with increased M1 polarization (rise in iNOS+IBA1+ cells) and elevated pro-inflammatory cytokines (IL-1β, TNF-α). Recombinant *IGF-1* (rIGF-1) and epigallocatechin gallate (EGCG) treatment restored *IGF-1* expression to 85% of normal levels, reduced M1 markers, and enhanced M2 markers through IGF-1 receptor (IGF1R)-mediated *JNK* inhibition and *STAT6* activation [56].

IL-35 therapy rebalanced microglial polarization in DNP models, decreasing Iba1+ cluster of differentiation 68 (CD68+) cell density while elevating CD206+ cells. Mechanistic studies revealed IL-35 suppresses *JNK* activation and enhances *JAK2/STAT6* phosphorylation, shifting cytokine profiles from M1-dominant (TNF-α, IL-6 decrease) to M2-skewed (IL-10). Pharmacological validation confirmed pathway specificity, with *JNK* activator anisomycin reversing M2 polarization effects and *JAK2* inhibitor AG490 blocking 76% of IL-35-mediated therapeutic outcomes [38].

### 4.2. Metabolic Regulation Pathways

*Sirtuin 3* (*Sirt3*), a mitochondrial deacetylase pivotal in cellular metabolism and oxidative stress regulation, attenuates microglial pro-inflammatory states by suppressing glycolysis and inflammatory signaling. The downregulation of *Sirt3* under hyperglycemic conditions exacerbates glycolytic metabolic reprogramming during diabetic neuropathic pain (DNP) pathogenesis. In Sirt3-deficient DNP mice, elevated expression of key glycolytic enzymes—including *hexokinase 2* (*HK2*), *pyruvate kinase M2* (*PKM2*), and *lactate dehydrogenase A* (*LDHA*)—was observed, accompanied by increased accumulation of glycolytic metabolites (lactate and pyruvate). Conversely, *Sirt3* overexpression in BV-2 microglia significantly reduced the expression of these enzymes (*p* < 0.01) and diminished metabolite levels, thereby mitigating pro-inflammatory activation. Mechanistically, *forkhead box O1* (*FoxO1*) serves as a critical mediator of *Sirt3*-dependent glycolytic control. While *FoxO1* overexpression suppressed glycolytic enzyme expression under normal conditions, this inhibitory effect was abolished in *Sirt3*-deficient microglia, indicating *Sirt3*-dependent regulatory function. Furthermore, protein kinase B (*Akt*) pathway activation promotes *FoxO1* inactivation and accelerates *Sirt3* degradation via autophagy-lysosome pathway (ALP)-mediated proteolysis, ultimately driving microglial glycolysis and neuroinflammation [36].

The inositol-requiring enzyme 1 alpha–X-box binding protein 1 (*IRE1α–XBP1s*) axis, a central mediator of endoplasmic reticulum stress (ERS) responses, is hyperactivated in microglia under hyperglycemic conditions. High glucose exposure upregulated prostaglandin-endoperoxide synthase 2 (*PTGS2*), *IRE1α*, and *XBP1s* expression in OX-42-positive microglia, concomitant with elevated secretion of TNF-α, IL-1β, and IL-6. Sinomenine (SIN) treatment reversed these effects, reducing *PTGS2* levels and suppressing *IRE1α–XBP1s* signaling through MKC8866 (*IRE1α* inhibitor), which subsequently decreased inflammatory cytokine release. Notably, *PTGS2* overexpression exacerbated inflammation, an effect counteracted by MKC8866. These findings collectively demonstrate that SIN alleviates diabetic peripheral neuropathic pain by downregulating *PTGS2* to inhibit the *IRE1α–XBP1s* pathway, thereby reducing microglial activation and neuroinflammation [57].

### 4.3. Neuroprotection and Repair

*Notch receptor 1* (*Notch-1*) signaling in microglia critically mediates the progression of diabetic neuropathy. Pharmacological inhibition of *Notch-1* using DAPT and/or minocycline effectively suppressed microglial proliferation and alleviated thermal hyperalgesia and mechanical allodynia in PDN models. Chronic administration of minocycline or DAPT for two weeks significantly attenuated PDN-associated mechanical withdrawal threshold (MWT) and thermal withdrawal latency (TWL) impairments (*p* < 0.01 vs. vehicle). This analgesic effect correlated with reduced *Notch-1* receptor expression, diminished nuclear translocation of the *Notch-1* intracellular domain, and suppressed *hairy and enhancer of split 1* (*Hes-1*) activation. Concurrent downregulation of Iba-1 expression confirmed *Notch-1*-dependent microglial activation and neuroinflammation. Streptozotocin (STZ)-induced diabetes upregulated *Notch-1* receptor expression and enhanced Notch intracellular domain (NICD)/Hes-1 signaling, indicating pathological activation of Notch signaling in diabetic neuropathy. These findings align with established mechanisms whereby Notch pathway activation exacerbates neural damage, while its inhibition prior to pain onset delays neuropathic progression [58].

### 4.4. Chemokine-Receptor Interactions

#### 4.4.1. *CXCL13/CXCR5* Axis

In PDN rat models, spinal dorsal horn microglia exhibited marked activation as evidenced by increased Iba-1 and cluster of differentiation 11b (CD11b) expression. Microglial activation coincided with *CXCL13/CXCR5* axis hyperactivation, where neuron-derived *CXCL13* binds microglial *CXCR5* receptors to potentiate neuroinflammatory responses. Exogenous *CXCL13* administration elevated spinal TNF-α, IL-6, and IL-1β levels in wild-type mice, effects abolished in *CXCR5*-knockout models. Immunohistochemical analysis revealed co-localization of *CXCR5* with Iba-1+ microglia in PDN spinal cords, with less than co-expression in neuronal nuclear protein (NeuN+) neurons. This pathway regulates mechanical hypersensitivity through microglial-mediated inflammatory cascades, as *CXCR5* deficiency reduced mechanical pain thresholds by and attenuated microglial activation [42].

#### 4.4.2. *XCL1/XCR1* Signaling

Microglial suppression attenuates X-C motif chemokine ligand 1/X-C motif chemokine receptor 1 (*XCL1/XCR1*)expression and exerts analgesic effects in streptozotocin (STZ)-induced PDN. Chlorpromazine (MC)-mediated microglial inhibition significantly reduced mechanical allodynia and dysesthesia in PDN models, concurrently preventing microglial activation and suppressing upregulation. Exogenous *XCL1* administration enhanced nociceptive transmission, whereas *XCL1*-neutralizing antibodies attenuated hypersensitivity. Primary microglial cultures demonstrated that activation triggers *XCL1* release and *XCR1* upregulation. Immunofluorescence localized *XCR1* predominantly to spinal neurons rather than astrocytes. In DNP progression, elevated *XCL1* levels drive neuronal *XCR1* overexpression, facilitating pain signal transduction. MC treatment reduced spinal *XCL1*, subsequently decreasing *XCR1* expression and alleviating pain behaviors. Notably, MC administration also diminished CD4/CD8 protein levels, suggesting immunomodulatory mechanisms underlying its analgesic efficacy [59].

#### 4.4.3. *FKN/CX3CR1* Axis

The fractalkine (*CX3CL1*)/*CX3CR1* axis, involving neuron/astrocyte-derived *CX3CL1* and microglial *CX3CR1* receptors, mediates neuropathic pain through pro-inflammatory activation. Diabetic mice exhibited increased *CX3CL1* and *CX3CR1* expression in spinal dorsal horn microglia. Intrathecal *CX3CR1*-neutralizing antibodies delayed mechanical pain onset and reduced pain severity scores in STZ models. *CX3CR1*-knockout mice demonstrated attenuated IL-1β and TNF-α levels, confirming this pathway’s role in microglial-mediated neuroinflammation. Mechanistically, *CX3CL1* binding induces microglial proliferation and chemotaxis, while promoting inflammatory mediator release. Paradoxically, *CX3CR1* deficiency correlates with impaired T-cell responses, suggesting bidirectional neuroimmune interactions in diabetic pain pathogenesis [35].

### 4.5. Additional Regulatory Mechanisms

#### 4.5.1. miR-23a Signaling

Low-concentration bupivacaine demonstrated superior efficacy in alleviating mechanical allodynia and thermal hyperalgesia in diabetic mice compared to higher doses. This analgesic effect correlated with microglial inflammation suppression, evidenced by reduced spinal TNF-α, IL-6, IL-1β, and MCP-1 levels. Mechanistically, bupivacaine upregulated microRNA-23a (miR-23a) expression in microglia, which directly targeted the 3’untranslated region (3′-UTR) of phosphodiesterase 4B (PDE4B) mRNA, achieving *PDE4B* downregulation. miR-23a knockdown using antagomirs abolished bupivacaine’s anti-inflammatory effects, establishing miR-23a as the pivotal mediator [60].

#### 4.5.2. Epigenetic Regulation

Diabetic neuropathic pain induces cell-type specific epigenetic modifications, with spinal microglia exhibiting increased *H3K9 acetylation* (*H3K9ac*) compared to neurons. Chromatin immunoprecipitation revealed HMGB1 as the primary histone acetyltransferase regulator in non-neuronal cells. Glycyrrhizin (GLC) treatment reduced High-Mobility Group Box 1 (*HMGB1*)release and normalized microglial *H3K9ac* levels, attenuating neuroinflammation through acetylation-dependent chromatin remodeling [34].

#### 4.5.3. MOTS-c (Mitochondrial-Derived Peptide)

The mitochondrial-encoded peptide MOTS-c ameliorates painful diabetic neuropathy via dual metabolic-inflammatory modulation. MOTS-c administration significantly reduced Iba1+ microglial density, concurrently decreased pro-inflammatory mediators including TNF-α, IL-1β, IL-6, and *iNOS*, while upregulating anti-inflammatory *Arg-1* expression. In vitro, MOTS-c suppressed LPS-induced microglial activation, lowering TNF-α/IL-6/IL-1β secretion while enhancing Arg-1 production. This anti-polarization effect persisted for 72 h post-treatment, confirming sustained therapeutic potential [61].

#### 4.5.4. *ADAM17* (A Disintegrin and Metalloproteinase 17)

*ADAM17*, an enzyme primarily responsible for membrane protein degradation, is activated under diabetic conditions and plays a critical role in painful diabetic neuropathy (PDN). In db/db diabetic mice, *ADAM17*-positive neurons significantly increased in spinal dorsal horn lamina IV, while *ADAM17*-positive microglia were elevated only in laminae I-II. Strong *ADAM17* expression was observed in NeuN-positive neurons and IBA1-positive microglia, with negligible localization in GFAP-positive astrocytes. Comparative analysis revealed a marked increase in *ADAM17*-positive neurons across laminae I-V of the db/db spinal dorsal horn, whereas *ADAM17*-positive microglia accumulation remained restricted to laminae I-II [62].

#### 4.5.5. H_2_S (Hydrogen Sulfide) Donor

Hydrogen sulfide (H_2_S), the third endogenous gasotransmitter following nitric oxide and carbon monoxide, exhibits diverse physiological effects and pathological associations. In STZ-induced diabetic rats, chronic administration of the H_2_S donor GYY4137 improved neuropathological indices and behavioral manifestations. Specifically, GYY4137 reduced spinal microglial activation (evidenced by decreased Iba-1+ cell counts and protein expression), attenuated pro-inflammatory cytokine levels, and ameliorated allodynia, mechanical hyperalgesia, and thermal hypersensitivity. These findings suggest that GYY4137 may represent a potential therapeutic strategy worthy of further investigation for diabetic neuropathic pain through dual modulation of central glial cells—suppressing microglia-mediated inflammation while regulating astrocytic metabolic support [63].

#### 4.5.6. *APPL1* Signaling Pathway

In PDN rats, *APPL1* protein expression progressively declined in the spinal dorsal horn from week 1, achieving a significant reduction by week 4 (*p* < 0.05), concurrent with decreased mechanical withdrawal thresholds (lowest at week 4 via von Frey testing) and shortened thermal withdrawal latencies. Immunofluorescence localization demonstrated higher *APPL1* expression in neurons and microglia versus astrocytes in controls, whereas PDN rats exhibited pronounced *APPL1* reduction in dorsal horn laminae I-II. This reduction correlated with upregulated *p-mTOR* expression (*p* < 0.05), indicating an inverse regulatory relationship between *APPL1* and *mTOR* activity. Furthermore, *APPL1* deficiency enhanced *Rab5*, *Akt*, and *AMPK* phosphorylation, suggesting *mTOR* activation through *Rab5/Akt* and *AMPK* pathways exacerbates hyperalgesia [45].

### 4.6. Limitations and Translational Considerations

The signaling pathways summarized herein, including *NLRP3*, *JAK/STAT*, and metabolic reprogramming, are primarily derived from rodent models. While offering crucial mechanistic insights, these findings are constrained by notable limitations. Pathophysiological differences between STZ-induced (T1DM) and *db/db* (T2DM) models may lead to divergent glial responses. Furthermore, while interventions like IL-35 and *MOTS-c* show promise preclinically, significant translational hurdles remain regarding their human safety, efficacy, and feasible delivery routes (e.g., intrathecal administration) [61]. Additionally, many pathways are reported by a limited number of studies, underscoring the need for independent validation in models that better mimic chronic human disease and for exploration of synergies with existing clinical agents like metformin.

Furthermore, when translating preclinical findings to humans, the heterogeneity of diabetes types must be taken into account. Classical studies indicate that the pathology of “axon-glial dysfunction” modeled in STZ-induced T1DM rats is reflected in human T1DM. In contrast, morphological manifestations of neuropathy in T2DM patients are often dominated by Wallerian degeneration and ischemic patterns [64]. This suggests that glial responses observed in T2DM models such as *db/db* mice may be compounded by complex factors including aging and vasculopathy. Future studies need to validate these findings in models that more closely mirror the complex pathophysiology of human T2DM.

Specifically, the acute hyperglycemia and insulin deficiency in STZ (T1DM) models may elicit a different neuroinflammatory profile compared to the chronic metabolic syndrome (insulin resistance, hyperlipidemia) in *db/db* (T2DM) models, which could differentially impact microglial activation states and therapeutic responses.

## 5. Astrocytes: Metabolic Collapse and Inflammatory Cycling

### 5.1. Impaired Metabolic Clearance and Neuroinflammation

#### 5.1.1. *AQP4* Function in Astrocytes

Studies demonstrate that astrocyte activation in PDN rats correlates with altered aquaporin-4 (*AQP4*) polarization, impairing spinal glymphatic metabolic waste clearance. PDN models exhibit astrocytic activation accompanied by depolarized *AQP4* distribution, manifested as reduced *AQP4* expression (fluorescence labeling quantification) and compromised waste removal. This dysfunction coincides with increased *MMP-9* and *NLRP3* levels, suggesting *AQP4* polarization defects constitute a key pathological mechanism in PDN. Restoring *AQP4* polarity or suppressing astrocyte overactivation may alleviate neuroinflammation and chronic pain [39].

The *NLRP3* inflammasome exacerbates neuroinflammation and chronic pain through astrocyte-mediated activation in PDN. *NLRP3* upregulation strongly associates with astrocyte activation, showing marked elevation in PDN progression. Bidirectional interactions exist between *AQP4* depolarization and *NLRP3* activation: *AQP4* dysfunction potentiates *NLRP3* inflammasome activity via impaired waste clearance and amplified neuroinflammatory cascades, thereby intensifying pain and neural damage [39].

*AQP4* depolarization, *MMP-9* elevation, and *NLRP3* activation form a self-perpetuating cycle in PDN, progressively aggravating neuroinflammation and chronic pain. Mechanistically, *AQP4* defects impair metabolic clearance while promoting *NLRP3*-driven inflammation, establishing this triad as pivotal therapeutic targets [39].

Beta-hydroxybutyrate (BHB) mitigates PDN by restoring *AQP4* polarity in spinal glymphatic systems. Emerging evidence indicates astrocytic endfoot-localized *AQP4* governs CNS metabolic clearance, with *syntrophin-α1* (*SNTA1*) anchoring *AQP4* to maintain membrane polarity. BHB elevates *SNTA1* expression to rescue *AQP4* polarization in Alzheimer’s models. This study pioneers BHB’s therapeutic potential for PDN through spinal glymphatic *AQP4* repolarization [43].

PDN rats exhibit glymphatic dysfunction characterized by a 40% reduction in metabolic clearance compared to controls, accompanied by enhanced mechanical allodynia (*p* < 0.01), 62% decreased *SNTA1* expression, and paradoxical *AQP4* upregulation with loss of perivascular polarity. Notably, beta-hydroxybutyrate (BHB) treatment reverses these pathological alterations, restoring *AQP4* protein levels, recovering 75% of *SNTA1* expression, and reestablishing vascular-polarized *AQP4* localization, thereby demonstrating therapeutic efficacy in glymphatic system rehabilitation [43].

In PDN rats, spinal glymphatic dysfunction manifests as impaired metabolic waste clearance, exacerbated mechanical allodynia, reduced *SNTA1* expression, elevated *AQP4* levels, and reversed *AQP4* polarity characterized by diminished perivascular localization. Compared to controls, PDN rats exhibited increased *AQP4* protein expression with concurrent *SNTA1* downregulation. BHB treatment reversed these alterations, normalizing *AQP4* expression, restoring *SNTA1* levels, and reestablishing vascular-polarized *AQP4* distribution [43].

*AQP4* polarity refers to the highly enriched distribution of this water channel protein on the astrocytic end-foot membranes, where it is anchored to the basement membrane of the perivascular space. This polarized spatial arrangement forms the structural basis for the efficient cerebrospinal–interstitial fluid exchange and metabolic waste clearance performed by the central nervous system’s “glymphatic system” [27,43]. In PDN, factors such as hyperglycemia drive the upregulation of proteolytic enzymes like matrix metalloproteinase-9 (*MMP-9*), leading to the cleavage of β-dystroglycan—a key protein that anchors *AQP4* at the astrocytic end-feet [39,43]. This anchorage disruption results in the loss of *AQP4* polarity, characterized by the dissociation of *AQP4* from end-foot membranes and its diffuse redistribution across the entire astrocytic plasma membrane. The reason this event is pivotal in initiating pathogenesis is that it directly dismantles glymphatic clearance capacity, leading to the accumulation of excitatory neurotransmitters (e.g., glutamate), inflammatory factors, and damaging metabolites in the spinal dorsal horn. This accumulation not only triggers direct neuronal excitotoxicity (via *NMDA* receptor hyperactivation) but also creates and sustains a pro-inflammatory, metabolically dysregulated microenvironment, thereby exacerbating neuroinflammation and central sensitization in a positive-feedback manner [40,43]. Therefore, *AQP4* polarity loss is not merely an epiphenomenon but constitutes a central hub event that links metabolic dysregulation (hyperglycemia), astrocytic dysfunction, and neuropathic pain (central sensitization).

#### 5.1.2. Astrocytic Involvement in Neuroinflammation

Beta-hydroxybutyrate (BHB), a class I histone deacetylase (HDAC) inhibitor and ketone body metabolite produced during caloric restriction, demonstrates anti-inflammatory effects across metabolic disorders including Parkinson’s disease, Alzheimer’s disease, and diabetic microvascular complications. Experimental evidence confirms BHB’s capacity to enhance *SNTA1* expression and restore *AQP4* polarity, suggesting therapeutic potential for PDN through glymphatic modulation [41].

Curcumin administration (50 mg/kg for 4 weeks) alleviates neuropathic pain by suppressing phosphorylated c-Jun N-terminal kinase (pJNK) signaling in diabetic models. Dorsal root ganglia analysis revealed elevated pJNK expression in astrocytes and neurons, which curcumin treatment effectively reduced. Immunohistochemical co-localization identified pJNK-positive astrocytes (GFAP+) and neurons (NF200+), with curcumin decreasing their prevalence, indicating dual cellular targeting in pain regulation [65].

The *CXCL13/CXCR5* axis predominantly activates spinal astrocytes in PDN, with *CXCR5* overexpression correlating with astrocytic activation. This pathway promotes neuroinflammation via downstream pERK and pSTAT3 signaling. Pharmacological inhibition of *CXCL13/CXCR5* attenuates astrocyte-mediated inflammatory responses and neuropathic pain, though precise mechanisms regulating cytokine release require further investigation [66].

### 5.2. Cellular Connectivity and Barrier Restoration

#### 5.2.1. Connexin 43 (Cx43) in Astrocytes: Implications for Neuropathic Pain

Connexin 43 (Cx43), predominantly expressed in central nervous system (CNS) astrocytes, plays critical roles in intercellular communication. By forming gap junctions, Cx43 facilitates signal transmission between cells, mitigates extracellular ionic imbalances, and supports metabolic coordination across neighboring cells. Each gap junction channel comprises two connexons, with each hexameric connexon assembled from six Cx43 subunits, underscoring its centrality in cellular signaling and metabolic regulation [67].

In neuropathic pain pathogenesis, astrocytic Cx43 demonstrates dual functionality in both pain maintenance and transduction. Partial sciatic nerve ligation (PSNL) rat models revealed significant Cx43 downregulation in the spinal dorsal horn by day 7 post-injury, despite early mechanical hypersensitivity observed at day 3. This temporal dissociation highlights Cx43’s pivotal role in sustaining chronic pain states. Restoring physiological Cx43 levels alleviates hypersensitivity, further validating its mechanistic importance in neuropathic pain perpetuation [67,68].

Lycopene administration reverses tumor necrosis factor (TNF)-induced Cx43 suppression, restoring spinal astrocytic Cx43 expression and ameliorating mechanical hypersensitivity. Notably, chronic intrathecal lycopene delivery—but not single-dose treatment—significantly elevates dorsal horn Cx43 levels and attenuates mechanical allodynia in peripheral neuropathic pain models. These findings position Cx43 expression modulation as a novel therapeutic strategy for neuropathic pain management, with potential translational relevance to painful diabetic neuropathy (PDN) treatment [67].

Under the chronic stress of PDN, the function of astrocytic Cx43 undergoes a pathological shift. On one hand, gap junctions formed by Cx43 may facilitate the spread of pro-nociceptive signals within the astrocytic network, thereby expanding the zone of inflammation. On the other hand, and more critically, the aberrant opening of Cx43 hemichannels leads to the direct release of ATP and inflammatory cytokines (e.g., IL-1β, TNF-α) into the extracellular space. These “danger signals” can directly activate purinergic receptors and inflammatory pathways on neighboring neurons and microglia, thereby enhancing neuronal excitability and sustaining the neuroinflammatory state, which in turn exacerbates hyperalgesia through a positive-feedback loop. Consequently, the acquired functional abnormalities of Cx43, rather than simple changes in its expression level, constitute a crucial link connecting astrocytic reactivity to neuronal hyperexcitability.

#### 5.2.2. *Matrix Metalloproteinase-9* (*MMP-9*) Mechanism

Astrocytes critically maintain neuroenvironmental homeostasis through metabolic waste clearance mediated by endfeet-localized *aquaporin-4* (*AQP4*). Under diabetic neuropathic pain (PDN) conditions, hyperglycemia induces *MMP-9* overexpression, triggering β-dystroglycan (β-DG) cleavage and impairing *AQP4* membrane anchoring. This pathological cascade disrupts metabolic waste clearance, exacerbating neuroinflammation and potentiating pain hypersensitivity [39].

### 5.3. Epigenetic and Transcriptional Regulation

#### 5.3.1. *HDAC5-STAT3* Pathway

Spinal astrocytic degeneration emerges prominently in type 1 diabetic (T1D) rat models during early-stage PDN progression. Hyperglycemia-induced astrocytic degeneration correlates with impaired *STAT3* acetylation, a transcription factor essential for astrocytic homeostasis. *STAT3* dysfunction precipitates functional loss, driving sustained pain hypersensitivity [41].

Mechanistically, *histone deacetylase 5* (*HDAC5*) hyperactivation in diabetic astrocytes mediates *STAT3* deacetylation via direct protein–protein interaction. This post-translational modification disrupts *STAT3* signaling capacity, compromising astrocytic structural integrity and neuroprotective functions. Restoring *STAT3* activity or pharmacologically inhibiting *HDAC5* ameliorates astrocytic degeneration and attenuates PDN symptoms. These findings delineate the *HDAC5-STAT3* axis as a pivotal regulator of diabetic astrocytopathy, providing novel therapeutic targets for PDN management [41].

#### 5.3.2. miR-503-5p/*SEPT9* Axis

The miR-503-5p/*SEPT9* axis suppresses astrocytic activation to alleviate PDN-associated pain. Emerging evidence highlights microRNAs as pivotal regulators and potential therapeutic targets in PDN pathogenesis. miR-503-5p specifically mitigates neuropathic pain in type 2 diabetic (T2DM) models by targeting *SEPT9* to inhibit astrocyte activation [69].

In db/db PDN mice, spinal miR-503-5p expression was significantly downregulated compared to db/m controls, correlating with hyperglycemia, mechanical allodynia (reduced mechanical withdrawal threshold), thermal hyperalgesia (shortened thermal withdrawal latency), and elevated GFAP/MCP-1 levels indicative of astrocytic activation. Parallel in vitro studies under high-glucose conditions recapitulated these findings, showing miR-503-5p suppression alongside astrocyte activation and increased IL-1β, IL-6, and TNF-α production. *SEPT9* was aberrantly upregulated in both diabetic mice and glucose-stimulated astrocytes, whereas miR-503-5p agomir treatment normalized *SEPT9* expression, reduced GFAP/MCP-1 levels, and improved pain thresholds. Conversely, antagomiR-503-5p exacerbated pain hypersensitivity and further elevated *SEPT9* [69].

Rescue experiments confirmed *SEPT9* as the mechanistic mediator: *SEPT9* overexpression in miR-503-5p mimic-treated cells reversed the suppression of GFAP, MCP-1, and pro-inflammatory cytokines, demonstrating *SEPT9*’s essential role in miR-503-5p-mediated analgesia. These results establish miR-503-5p/*SEPT9* signaling as a critical regulator of astrocyte-driven neuropathic pain in DPN, highlighting its therapeutic potential [69].

### 5.4. Activation of Astrocytes in the Ventrolateral Periaqueductal Gray (vlPAG)

During PDN progression, astrocytes in the ventrolateral periaqueductal gray (vlPAG) exhibit marked proliferation and morphological alterations, with activation initiating at day 14 and peaking by day 21, characterized by somatic hypertrophy, process elongation, and increased branching [70].

Chemogenetic activation of vlPAG astrocytes via Gq-DREADDs induced neuropathic pain behaviors and pain-related aversion, indicating their promotive role in PDN. Conversely, Gi-DREADDs-mediated inhibition alleviated pain hypersensitivity, confirming vlPAG’s critical involvement in nociceptive processing. Modulation of vlPAG astrocytic activation may offer novel therapeutic avenues for PDN management [70].

Fluorocitrate (FC) and neurotrophins demonstrated analgesic effects in PDN by suppressing vlPAG astrocyte activation. Compared to controls, PDN rats showed elevated GFAP expression in vlPAG (*p* = 0.002) and reduced mechanical withdrawal thresholds (MWT). Local FC microinjection or systemic neurotrophin administration increased MWT while decreasing GFAP+ cell density and protein levels (*p* < 0.001), suggesting astrocyte-targeted analgesia [71].

### 5.5. Astrocytic Neuroprotection Mechanisms

In type 2 diabetic PDN, astrocyte activation correlates with *NMDAR*-dependent signaling. Ten-week-old db/db mice exhibited increased substance P (SP) expression and enhanced *ERK1/2* phosphorylation in lumbar spinal cord dorsal horn (LSCDH) layers I-III, accompanied by astrocytic hyperplasia. Concurrent *NMDAR* (*NR1* subunit) phosphorylation upregulated neuronal *nNOS* and astrocytic *iNOS*, driving nitric oxide (NO) cascade activation. Pharmacological inhibition with MK801 (*NMDAR* antagonist) reduced *nNOS/iNOS* expression, attenuated GFAP+ astrocyte activation, and ameliorated mechanical allodynia. L-NAME (NOS inhibitor) and UO126 (*ERK1/2* inhibitor) produced comparable anti-nociceptive effects, confirming the *NMDAR-NO-ERK1/2* axis as a central pathway in PDN pathogenesis [40].

### 5.6. MCx Region Astrocyte Activation and Pain Modulation

Astrocytes regulate motor cortex (MCx) neuronal activity via pro-inflammatory cytokine release (e.g., *TNF-α*, *IL-1β*), amplifying pain perception in DNP. Elevated GFAP expression in PDN rat MCx regions implicates astrocytic activation in neuropathic pain maintenance. Chemogenetic inhibition of MCx excitatory neurons alleviated mechanical allodynia, whereas their activation exacerbated pain. Astrocyte-neuronal crosstalk potentiates nociceptive responses, with *TNF-α/IL-1β* serving as key mediators sustaining neuropathic pain. Targeting astrocytic activation or cytokine release may yield novel therapeutic strategies for PDN [72].

### 5.7. Limitations and Translational Considerations

The pathological mechanisms of astrocytes summarized in this section, such as *AQP4* polarity loss, Cx43 dysfunction, and the dysregulation of signaling pathways like *HDAC5-STAT3*, are primarily derived from rodent models like STZ-induced or *db/db*. While these models provide crucial insights into astrocytic dysfunction in PDN, caution is required when extrapolating findings to human disease. Differences exist between STZ (T1DM-like) and *db/db* (T2DM-like) models regarding the origin of metabolic disturbances, progression rate, and systemic complications, which may influence the spatiotemporal characteristics and severity of astrocytic responses. For instance, hyperglycemia-induced *MMP-9* overexpression and β-dystroglycan cleavage may not be entirely equivalent between the two models. Furthermore, most in vitro studies employ primary astrocytes or cell lines cultured under high glucose conditions, a simplified system that cannot fully replicate the complex microenvironment of the neurovascular unit in vivo or the dynamic interactions with neurons, microglia, and oligodendrocytes. Therefore, when interpreting the contribution of specific pathways (e.g., *CXCL13/CXCR5* or miR-503-5p/*SEPT9*), their experimental model context must be considered. Future research should utilize models that more closely mimic the chronic progression of human disease (e.g., diet-induced combined with low-dose STZ T2DM models) and incorporate humanized astrocyte or organoid models to validate the translational potential of these targets and elucidate the specificity of astrocytopathy in different types of diabetes.

## 6. Oligodendrocytes: Overlooked Modulators of Pain in PDN

The role of oligodendrocytes in PDN remains underexplored, with current evidence being preliminary and largely derived from a limited number of preclinical studies. Study reveals a significant increase in oligodendrocyte numbers in the spinal dorsal horn of PDN rats, accompanied by hypermyelination in the spinothalamic tract. These findings suggest that dysmyelination or structural myelin abnormalities may contribute to central sensitization. Metformin treatment alleviates pain by suppressing excessive myelination, though its precise mechanism remains unclear [45]. Further analysis identifies that *APPL1* deficiency exacerbates oligodendrocyte dysfunction through *mTOR* signaling activation and *Rab5* upregulation, leading to demyelination and hyperalgesia, while *APPL1* overexpression reverses these effects [45]. These results highlight the critical link between oligodendrocytic myelin homeostasis and the *mTOR/Rab5* pathway, suggesting its potential role in sustaining PDN-related pain.

Oligodendrocyte precursor cells (OPCs) may influence pain transmission via paracrine mechanisms due to their aberrant proliferation. Study reports that OPCs secrete the chemokine *CCL2*, which activates *CCR2* receptors on spinal dorsal horn neurons to directly amplify nociceptive signaling [45]. Although the exact pathways remain unelucidated, the pro-inflammatory role of the *CCL2/CCR2* axis in other neuropathies supports its potential involvement in PDN, possibly through indirect activation of microglia or neuronal *NMDA* receptors. Additionally, interactions between oligodendrocytes and astrocytes—such as metabolic coupling or gap junction communication—remain unexplored and represent a crucial area for future investigation.

Recent lipidomic studies have further revealed that in obesity-associated diabetic neuropathy, the loss of myelin-characteristic lipids (such as galactosylglyceramide) occurs prior to structural deficits. This process is closely associated with obesity/hyperlipidemia, suggesting that lipid metabolism disturbances may represent an important mechanism—distinct from hyperglycemia—that drives early damage to the oligodendrocyte-myelin axis [73]. These findings provide a more upstream metabolic basis for understanding myelin abnormalities in PDN. Although the study focused on peripheral nerves, the revealed lipid-myelin axis mechanism likely exerts parallel or interactive effects in the central nervous system, warranting validation in future PDN models.

**Figure 2:**
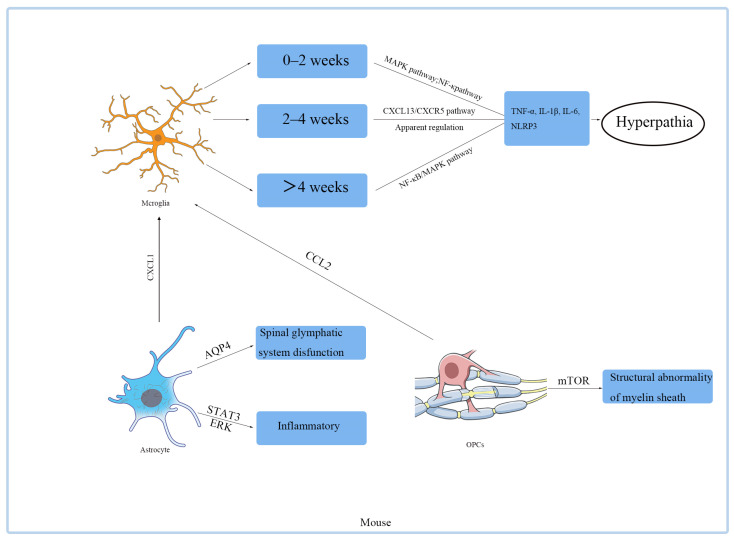
Pathological Roles of Glial Cells in Painful Neuropathy: Molecular Mechanisms and Signaling Pathways. Microglia: Activated by nerve injury or metabolic stress (e.g., diabetes), releasing pro-inflammatory cytokines (TNF-α, IL-1β, IL-6) and chemokines (e.g., *CCL2*). These mediators enhance neuronal excitability via *TLR4/NF-κB* and *p38 MAPK* pathways, driving central sensitization. Astrocytes: Respond to microglial signals (e.g., ATP, cytokines) through gap junctions, amplifying inflammation via *JAK-STAT* and *NF-κB* pathways. Astrocytic dysfunction disrupts glutamate homeostasis: specifically, functional abnormalities of connexin 43 (Cx43), such as excessive hemichannel opening, promote the release of pain mediators, while concurrently, the downregulation or impaired function of the glutamate transporter *GLT-1* directly impedes synaptic glutamate clearance. The resulting accumulation of glutamate leads to excessive activation of *NMDA* receptors, which is a key driver of central sensitization. Oligodendrocytes: Demyelination due to metabolic dysfunction (e.g., hyperglycemia) impairs axonal conduction, leading to ectopic discharges and pain. Key cross-talk: Bidirectional signaling between glia and neurons (e.g., via *CX3CL1-CX3CR1*) sustains chronic pain. Targeting glial activation (e.g., minocycline for microglia; fluorocitrate for astrocytes) may alleviate neuropathic pain. Abbreviations: *TLR4*, Toll-like receptor 4; *MAPK*, mitogen-activated protein kinase; *JAK-STAT*, Janus kinase-signal transducer and activator of transcription; *CX3CL1*, chemokine (C-X3-C motif) ligand 1.

Therapeutic strategies targeting oligodendrocytes remain exploratory. Study proposes that metformin may indirectly promote remyelination by modulating *mTOR* signaling or energy metabolism, though its specific targets require validation [45]. Building on the *APPL1/mTOR* axis, novel approaches such as *HDAC3* inhibitors (to enhance OPC differentiation) or neurotrophic factor interventions (to restore myelin integrity) could emerge as viable strategies for PDN management. Furthermore, integrating multi-omics technologies to delineate the transcriptomic and epigenetic profiles of oligodendrocytes across PDN stages may unravel precise mechanisms underlying their pain-regulatory functions (Figure 2).

Current understanding of the role of oligodendrocytes in PDN is still in its infancy, with related mechanisms based on limited studies and highly dependent on specific preclinical models. Existing evidence suggests that STZ and *db/db* models may exhibit different oligodendrocyte response patterns. Acute hyperglycemia induced by STZ may primarily trigger inflammation-mediated myelin damage, while the long-term metabolic syndrome background (e.g., insulin resistance, lipid metabolism abnormalities) in *db/db* models may be more likely to lead to energy metabolism disorder-related myelin maintenance defects and impaired regeneration. Findings such as the *APPL1-mTOR/Rab5* pathway and the *CCL2/CCR2* axis require independent validation in more diverse models (including female animal models to account for gender differences). It is particularly important that it remains unclear to what extent oligodendrocyte responses in the rodent spinal cord can mimic white matter pathology in the central nervous system (potentially involving higher brain regions) of human PDN patients. Targeting oligodendrocytes presents unique challenges: strategies aimed at promoting remyelination (e.g., *mTOR* modulators) must be precisely balanced to avoid aberrant hypermyelination potentially observed in the PDN context. Future research should employ longitudinal multimodal approaches (e.g., in vivo myelin imaging combined with molecular profiling) to systematically characterize the behavior of oligodendrocytes and their precursor cells at different pain stages in models that more closely reflect the pathophysiology of human T2DM, and to assess their interactions with astrocyte and microglial dysfunction, thereby identifying the most translationally promising timing and targets for intervention.

It is noteworthy that even in other diabetes-related neurological disorders with evident white matter involvement, the direct pathogenic role of oligodendrocytes remains inconclusive. For instance, in mouse and iPSC models of Wolfram syndrome—a condition characterized by diabetes and severe neurodegeneration—oligodendrocyte differentiation and core functions, such as mitochondrial-ER interactions, are largely preserved despite observed myelin abnormalities. This suggests that the myelination defects may arise from interactions with other cell types or systemic metabolic disturbances, rather than from autonomous oligodendrocyte dysfunction [74]. This finding further emphasizes that, in the context of painful diabetic neuropathy (PDN), it may be premature to directly equate initially observed changes in oligodendrocyte numbers or myelin-related molecules with definitive “driving” functional deficits in pain. Future research must move beyond correlative descriptions and strive to establish causal links between oligodendrocyte state alterations and pain behaviors in both in vivo and in vitro models, clarifying whether these cells act as “active players” or “secondary responders” within the interactive network involving microglia and astrocytes.

## 7. Biomarkers: From Bench to Bedside

Biomarker research in painful diabetic neuropathy (PDN) focuses on glia-specific molecules to distinguish peripheral and central pathological mechanisms. Microglia-associated biomarkers include cerebrospinal fluid (CSF)-soluble triggering receptor expressed on myeloid cells 2 (*TREM2*), which reflects phagocytic activity and correlates with neuroinflammatory burden, as well as *TSPO*-PET/MRI imaging—a noninvasive technique for mapping activated microglia in vivo [75]. Serum exosomal miRNAs, whose reduced levels inversely track microglial *NLRP3* inflammasome hyperactivity, may predict responsiveness to anti-inflammatory therapies [76]. Astrocyte-derived biomarkers emphasize *GFAP* (elevated CSF/serum concentrations indicating astrogliosis and metabolic dysfunction) and *AQP4* polarity imaging via high-resolution MRI, which visualizes glymphatic system impairment [77]. Oligodendrocyte biomarkers include myelin basic protein (MBP) for assessing demyelination and *CCL2/CCR2* axis activity, where elevated serum *CCL2* levels correlate with spinal oligodendrocyte precursor cell proliferation [16].

Translating glial biomarkers into clinical practice faces three major barriers: (1) Noninvasive detection limitations—*TSPO*-PET lacks the resolution to differentiate microglial subtypes, and *AQP4* polarity imaging requires ultra-high-field MRI (≥7T), hindering widespread adoption [15]. (2) Cross-species validation gaps—rodent models inadequately replicate human *CCL2/CCR2* signaling, necessitating humanized glial organoids for mechanistic validation [15]. (3) Dynamic monitoring standards—existing biomarkers like *GFAP* lack validated thresholds for disease staging, requiring longitudinal protocols such as serial CSF analysis paired with wearable pain sensors [22].

Emerging strategies aim to bridge biomarker discovery and targeted therapies: (1) Spatiotemporal biomarkers, such as *CX3CR1*-specific fluorescent nanoprobes, enable the real-time tracking of neuroinflammation by targeting live glial activation states [35]. (2) Closed-loop feedback systems, including implantable devices that release anti-inflammatory agents (e.g., IL-35) in response to biomarker detection (e.g., elevated TNF-α) [34]. (3) Patient stratification through combinatorial biomarkers (*TSPO* + *GFAP* + *MBP*) to define PDN subtypes, guiding personalized therapies—anti-inflammatory agents for microglia-dominant cases and remyelination promoters for oligodendrocyte pathology.

The growing understanding of glial dysregulation in PDN has unveiled a wealth of potential therapeutic targets. As detailed in Table 1, these strategies range from modulating microglial polarization and inflammasome activity, restoring astrocytic homeostasis and glymphatic function, to promoting oligodendrocyte integrity and remyelination. This consolidated view of glial cell-associated mechanisms and intervention strategies not only clarifies the pathophysiological contributions of each glial subset but also provides a rational basis for designing targeted therapies, potentially enabling personalized treatment approaches based on the predominant glial pathology in individual PDN patients.

While techniques such as TSPO-PET and *AQP4* polarity imaging hold significant promise, their clinical translation faces substantial hurdles, including high technical barriers and prohibitive costs associated with ≥7T MRI systems and specialized PET ligands, unresolved questions regarding their diagnostic specificity and sensitivity for PDN versus general neural injury or diabetes itself which require large-scale clinical cohort validation, and the considerable engineering, long-term biocompatibility, and regulatory challenges that place biomarker-responsive “closed-loop” therapeutic systems (e.g., for IL-35 release) far beyond current feasibility, making the more immediate path the development of readily detectable, blood-based biomarker panels (e.g., *GFAP*, *CCL2*) for patient stratification.

## 8. Concluding Remarks

This review cites numerous statistical metrics from primary preclinical studies. It is crucial to interpret these values—such as specific *p*-values or percentage changes—with caution, as they are highly dependent on the experimental context (e.g., model type, timing, dosage) and should not be directly extrapolated to human disease. As a narrative synthesis without meta-analysis, this work does not provide aggregate effect sizes or formal assessments of heterogeneity; the findings should therefore be regarded as important but preliminary mechanistic signals that require further validation.

By consolidating a substantial body of evidence, this review proposes the integrated “glial triad” framework as a central pathogenic nexus in PDN. This perspective moves beyond a neuron-centric view to highlight the functionally coupled, and often self-reinforcing, interactions among microglia, astrocytes, and oligodendrocytes across neuroimmune, metabolic, and structural axes. Synthesizing their interdependent roles provides a more holistic model for understanding the complexity of PDN and the phenomenon of central sensitization.

However, translating this mechanistic framework into clinical practice is fraught with significant challenges that must be rigorously acknowledged. Fundamental species differences between animal models and human patients, combined with the clinical infeasibility of invasive delivery routes (e.g., intrathecal) for many promising biologic agents, pose major translational hurdles. Furthermore, the long-term safety of modulating novel glial targets in humans remains completely unknown. Even advanced biomarker tools, such as *TSPO*-PET or ultra-high-field MRI for assessing glymphatic function, face prohibitive costs and technical barriers that limit their near-term clinical utility.

Therefore, future research must balance ambitious mechanistic discovery with pragmatic translational strategies. Priority should be given to repurposing widely used and safe agents (e.g., metformin) within this framework, developing accessible blood-based biomarker panels for patient stratification, and conducting rigorous early-phase trials for feasible interventions like beta-hydroxybutyrate supplementation. Concurrently, the field must employ advanced techniques to establish causal, spatiotemporally resolved links between glial states and pain phenotypes in models that better mimic human disease. In summary, while the “glial triad” model opens a transformative perspective on PDN pathogenesis, its ultimate value will be determined by a focused and critical effort to bridge the formidable gap between compelling preclinical insight and effective, safe, and accessible patient care.

## Figures and Tables

**Table 1:** Glial Cell-Associated Molecular Mechanisms and Therapeutic Strategies in Painful Diabetic Neuropathy (PDN).

Glial Cell Type	Key Molecules/Markers	Signaling Pathways	Pathological Role	Intervention Strategies	Reference
Microglia	*Iba-1*↑, TNF-α, IL-1β	p-p38/p-ERK/p-JNK, NF-κB	Early phase pain hypersensitivity	Ammoxetine (p-p38/p-JNK inhibitor)	[48]
	*NLRP3*↑, IL-1β↓	*NLRP3* inflammasome	Alleviation of thermal hyperalgesia	GLP-1RA (intracerebroventricular administration)	[50,51]
	*Sirt3*↓, *HK2*/*PKM2*↑	*Sirt3*-*FoxO1*-glycolysis axis	Enhanced glycolysis-driven inflammation	Metformin (Sirt3 upregulation)	[36]
	*CD206*↑, *Arg-1*↑	*JAK2/STAT6*↑, *JNK*↓	Anti-inflammatory M2 polarization	IL-35 injection	[38]
	JMT↓, p-STAT3↓	*JAK2/STAT3*	Neuroinflammation suppression	JMT (JAK2 inhibitor)	[53]
	*P2Y14*↑	*P2Y14-STAT1*/LncRNA-UC.25+	Enhanced inflammatory response	*P2Y14* shRNA	[54]
	miR-23a↑, PDE4B↓	miR-23a/PDE4B axis	Inhibition of cytokine release	Low-dose bupivacaine	[60]
	*XCL1/XCR1*↑	*XCL1/XCR1*-*p38 MAPK*	Enhanced pain transmission	*XCL1* neutralizing antibody	[59]
	*Notch-1*↓, *NICD*↓	*Notch-1* signaling	Suppression of microglial activation	Minocycline + DAPT (Notch inhibitor)	[58]
	*Ascl1/Lhx6*↑	*p38/JNK/NF-κB*↓	Reduced pro-inflammatory cytokines (TNF-α↓, IL-1β↓)	Intrathecal delivery of *Ascl1/Lhx6*	[49]
	*P2X7R*↑	*P2X7R* (microglia-specific)	Mechanical allodynia	*P2X7R* antagonist	[55]
	*TBK1*↑, GSDMD↑	*TBK1*-non-canonical NF-κB-*NLRP3*	Pyroptosis-driven chronic pain	*TBK1*-siRNA or AMX (*TBK1* inhibitor)	[37]
	*FKN/CX3CR1*↑	*FKN/CX3CR1-NLRP3*	Pro-inflammatory cytokine release	*CX3CR1* neutralizing antibody	[35]
	*CXCL13/CXCR5*↑	*CXCL13/CXCR5-pERK/pSTAT3*	Enhanced spinal inflammation	*CXCR5* gene knockout	[42,66]
	*HMGB1* acetylation↑, *H3K9ac*↑	*HMGB1-TLR4/NLRP3*	Epigenetic regulation of inflammation	Glycyrrhetinic acid (GLC)	[34]
	*MOTS-c*↑, *Arg-1*↑	*MOTS-c-HMGB1* axis	Inhibition of M1 polarization	MOTS-c therapy	[61]
	IGF-1↓, iNOS↑	IGF-1/IGF1R signaling	M1 polarization exacerbation	Recombinant IGF-1 (rIGF-1) or EGCG	[56]
	*ADAM17*↑	*ADAM17* (neuron/microglia)	Pro-inflammatory cytokine release	No specific targeted drug identified	[62]
	*BHB*↑, *SNTA1*↑	*BHB-SNTA1-AQP4* polarity restoration	Improved metabolic waste clearance	BHB supplementation	[43]
	*GYY4137*↑, *Iba-1*↓	*TLR9/p38 MAPK*↓	Inhibition of inflammation and glial activation	H_2_S donor GYY4137	[63]
	CD11b↑, TNF-α↓	Undefined	Gut-spinal inflammatory axis modulation	Ginger extract supplementation	[42]
	p-STAT3↑, p-CAV-1↑	*JAK2/STAT3-CAV-1-NR2B*	Enhanced NMDA receptor activation	AG490 (JAK2 inhibitor)	[53]
	*APPL1*↓, *p-mTOR*↑	*APPL1-mTOR/Rab5*	mTOR activation exacerbates hyperalgesia	*APPL1* overexpression	[45,46]
	*SEMA*↓, *IBA-1/GFAP*↓	*DPP-4* inhibition pathway	Suppression of glial activation	*DPP-4* inhibitor (Teneligliptin)	[33,36,42]
	*SIN*↓, *PTGS2/IRE1/XBP1s*↓	*PTGS2-IRE1α-XBP1s*	Inhibition of inflammatory cytokine secretion	Sinomenine (SIN)	[57]
	DEX↓, *Iba-1*↓	Undefined	Selective microglial inhibition	Dexmedetomidine (DEX)	[48]
	*TLR9*↑, p-p38/p-ERK↑	*TLR9*-*p38 MAPK/ERK1/2*	Enhanced inflammatory cytokine release	TLR9 shRNA or SB203580 (p38 inhibitor)	[51]
Astrocytes	*MMP-9*↑, *AQP4* polarity disruption	*MMP-9/β-DG/AQP4* axis	Impaired metabolic waste clearance	BHB (restores AQP4 polarity)	[39,40]
	*HDAC5*↑, *STAT3* deacetylation↓	*HDAC5-STAT3*	Astrocytic degeneration	*HDAC5* inhibitor	[41]
	*Cx43*↓	*Cx43* gap junction repair	Mechanical hypersensitivity alleviation	Lycopene	[67,68]
	miR-503-5p↓, *SEPT9*↑	miR-503-5p/*SEPT9* axis	Pro-inflammatory cytokine release (IL-1β↑, IL-6↑)	agomiR-503-5p	[69]
	*GFAP*↑, TNF-α/IL-1β↑	TNF-α/IL-1β-*MCx* neuron activation	Enhanced pain perception	Chemogenetic inhibition of *MCx* neurons	[72]
	vlPAG GFAP↑	Gq-DREADDs/Gi-DREADDs	Pain behavior modulation	DREADDs technology intervention	[71]
	*CXCR5*↑, pERK/pSTAT3↑	*CXCL13/CXCR5*-pERK/pSTAT3	Enhanced inflammatory response	*CXCR5* inhibitor	[42]
	*HMGB1*↑, *TLR4*↑	*HMGB1-RAGE/TLR4-NLRP3*	Aggravated neuroinflammation	GLC (HMGB1 inhibition)	[34]
	*NR1*↑, *ERK1/2*↑, iNOS↑	*NMDAR*-ERK1/2-NO	Astrocyte proliferation	MK801 (NMDAR inhibitor)	[51]
	*GFAP*↓, *NGF*↑	*NGF* signaling pathway	Inhibition of glial activation	Duloxetine	[55,62]
	*GFAP*↑ (restored)	H_2_S anti-inflammatory pathway	Improved metabolic support	GYY4137	[63]
	pJNK↓, *GFAP*↓	*JNK* signaling inhibition	Reduced DRG astrocyte activation	Curcumin	[65]
	vlPAG *GFAP*↓	Undefined	Increased mechanical withdrawal threshold	Fluorocitrate (FC) or neurotrophins	[71]
	*APPL1↓, mTOR*↑	*APPL1*-*mTOR/Rab5*	Functional disruption exacerbation	*APPL1* overexpression	[45]
Oligodendrocytes	*MBP*↑, *CCL2*↑, *APPL1*↓	*CCL2/CCR2* axis, *mTOR/Rab5*	Demyelination, OPC abnormal proliferation	Metformin (inhibits hypermyelination), *APPL1* overexpression	[16]

Symbols: ↑, upregulation/increase; ↓, downregulation/decrease. Statistical Significance: The effects reported in the table are derived from the cited original studies and are typically reported as statistically significant compared to controls (e.g., *p* < 0.05). For specific *p*-value ranges, please refer to the source articles. Data Source: The information in this table is synthesized from the preclinical studies (animal models and in vitro studies) cited in the main text. Abbreviations: Abbreviations not explicitly defined in the “Key Molecules/Markers” column can be found in the Abbreviations list at the end of the manuscript.

## Data Availability

No new data were created or analyzed in this study.

## Abbreviations

The following abbreviations are used in this manuscript:

AQP4	Aquaporin-4
Arg-1	Arginase-1
BHB	Hydroxybutyrate β
CCL2	C-C Motif Chemokine Ligand 2
CCR2	C-C Motif Chemokine Receptor 2
CSF	Cerebrospinal Fluid
Cx43	Connexin 43
DEX	Dexmedetomidine
DPP-4	Dipeptidyl Peptidase-4
EGCG	Epigallocatechin Gallate
ERK	Extracellular Signal-Regulated Kinase
GFAP	Glial Fibrillary Acidic Protein
GLP-1RA	Glucagon-Like Peptide-1 Receptor Agonist
GSDMD	Gasdermin D
HDAC5	Histone Deacetylase 5
HMGB1	High-Mobility Group Box 1
Iba-1	Ionized Calcium-Binding Adapter Molecule 1
IGF-1	Insulin-Like Growth Factor 1
IL-1β/IL-6/IL-10	Interleukin-1β/Interleukin-6/Interleukin-10
JAK2/STAT	Janus Kinase 2/Signal Transducer and Activator of Transcription 3
MAPK	Mitogen-Activated Protein Kinase
MBP	Myelin Basic Protein
MMP-9	Matrix Metalloproteinase-9
MOTS-c	Mitochondrial-Derived Peptide (Mitochondrial Open Reading Frame of the 12S rRNA)
mTOR	Mammalian Target of Rapamycin
NF-κB	Nuclear Factor Kappa-Light-Chain-Enhancer of Activated B Cells
NGF	Nerve Growth Factor
NLRP3	NLR Family Pyrin Domain Containing 3
NMDA/NMDAR	N-Methyl-D-Aspartate Receptor
OPC	Oligodendrocyte Precursor Cell
PDN	Painful Diabetic Neuropathy
PET-MRI	Positron Emission Tomography–Magnetic Resonance Imaging
SEMA	Teneligliptin (DPP-4 inhibitor)
Sirt3	Sirtuin 3
TLR4/TLR9	Toll-Like Receptor 4/9
TNF-α	Tumor Necrosis Factor-α
TSPO	Translocator Protein (18 kDa)
